# Successful use of regional anesthesia in non-intubated video-assisted thoracic surgery in patients with cardiopulmonary failure: two case reports

**DOI:** 10.1186/s40981-018-0183-0

**Published:** 2018-06-18

**Authors:** Satoru Kayama, Haruna Yamamoto, Shigehito Sawamura

**Affiliations:** Department of Anesthesiology, Teikyo University School of Medicine, 2-11-1 Kaga, Itabashi, Tokyo, 173-8605 Japan

**Keywords:** Non-intubated, Regional anesthesia, Paravertebral block (PVB), Video-assisted thoracic surgery (VATS), Cardiopulmonary failure

## Abstract

**Background:**

One-lung ventilation under general anesthesia is necessary for thoracic surgery, but this procedure is often difficult in surgery for patients with cardiopulmonary failure. Non-intubated video-assisted thoracic surgery (VATS) is performed under local anesthesia for patients with respiratory failure, but has not been performed for patients with circulatory failure. Here, we report management of two patients with cardiopulmonary failure who underwent non-intubated VATS with paravertebral block and infiltration anesthesia.

**Case presentation:**

Case 1 was a 79-year-old male with dyspnea at rest due to left large pleural effusion and cardiac dysfunction who underwent thoracoscopic pleural biopsy with paravertebral block under spontaneous breathing. The patient was also receiving dialysis. Case 2 was a 53-year-old male who developed empyema due to large pleural effusion, resulting in a poor general condition and cardiac dysfunction, and underwent video-assisted empyema curettage only with infiltration anesthesia under spontaneous breathing. In both patients, intraoperative respiration and circulation remained stable with values similar to those present preoperatively, and there were no problems after surgery.

**Conclusions:**

We safely anesthetized two patients with difficulty to general anesthesia by ensuring sufficient regional anesthesia during VATS under spontaneous breathing. These cases suggest that regional anesthesia for non-intubated VATS can contribute to maintain intra- and postoperative respiration and circulation in patients with cardiopulmonary failure.

## Background

Thoracic surgery is normally performed under general anesthesia because one-lung ventilation is necessary to ensure a wide visual field and pneumonectomy. However, some patients with comorbidities such as severe respiratory dysfunction cannot tolerate general anesthesia with one-lung ventilation, resulting in failure of surgical treatment. Non-intubated VATS performed under local anesthesia has fewer risks for pulmonary disorder caused by general anesthesia, such as barotrauma and atelectasis, due to use of intraoperative spontaneous breathing control, and this reduces postoperative respiratory complications [1, 2] and may allow patients with comorbidities to tolerate surgical treatment. Non-intubated VATS under local anesthesia may also be useful for patients with cardiac failure because it causes less cardiovascular restriction than general anesthesia, and local anesthesia causes no cardiovascular restriction; however, there are several case reports of patients with respiratory failure who underwent non-intubated VATS [1–7], but there is no case report of the use of the method in patients with respiratory failure complicated with unstable hemodynamics. Here, we report performance of regional anesthesia including paravertebral block and infiltration anesthesia for non-intubated VATS in two patients with severe respiratory and circulatory failure.

## Case presentation

### Case 1

A 79-year-old male (height 162 cm, weight 50.6 kg) was transferred to our hospital for diagnosis of the primary disease causing pleural effusion, and left thoracoscopic pleural biopsy was scheduled. Dyspnea developed due to left pleural effusion 1 month before surgery and pleural fluid aspiration was performed, but pleural effusion recurred several days after. Maintenance of blood pressure during dialysis treatment was not possible due to decreased circulating blood volume, and consequently, further pleural fluid aspiration was difficult. Respiration was shallow due to left pleural effusion, and SpO2 at rest was 91–95%. The patient also had cardiac dysfunction (ejection fraction (EF) 41%). Therefore, general anesthesia with one-lung ventilation was viable based on preoperative cardiopulmonary function, but there was a possibility of induction of postoperative cardiopulmonary insufficiency. Therefore, anesthetic management with dexmedetomidine (DEX) in combination with paravertebral block (PVB) was planned because the surgery was expected to be brief.

He also had angina and had started dialysis 26 years before. He underwent coronary artery bypass grafting and bypass grafting of the right axillary and bilateral femoral arteries due to arteriosclerosis obliterans (ASO). He was also complicated with moderate aortic stenosis (AS).

The patient was monitored with an electrocardiogram using five electrodes, a non-invasive arterial pressure meter and pulse oximeter, and an invasive arterial pressure line placed in the right radial artery. Jackson-Rees and nasal high flows were prepared for hypoxemia during surgery. In preparation for ventricular fibrillation, defibrillator pads were attached prior to surgery. The insertion point (T6) of the chest tube was confirmed and chest drainage was started at 0.8 μg/kg/h DEX without a loading dose. Ultrasound-guided PVB was performed with 0.5% mepivacaine (10 mL) and 0.375% ropivacaine (20 mL) at T6/7 with the patient in the lateral position. After confirming that the patient had a decreased cold sensation (T2-T9), surgery was performed and completed without intraoperative problems. SpO2 was maintained at > 90% with oxygen inhalation (6 L) by a face mask because of a potentially decrease in SpO2 by one-lung ventilation during the operation. The operative time was 39 min, and the anesthetic time was 67 min. The hemorrhage volume was small and the infusion volume was 260 mL. Intra- and postoperative changes in blood pressure were minor and similar to preoperative changes. After surgery, the patient was fully awake and returned to the ICU without oxygen supplementation. The patient was followed up without any problems. On postoperative day (POD) 1, the patient was moved out of the ICU and had no complications due to anesthesia. The patient was discharged from hospital on POD 7, but was readmitted on POD 23 due to hypokalemia-induced cardiac arrest. He was resuscitated from cardiac arrest, but he remained in a coma and died of multiple organ failure after pneumonia-induced septic shock on POD 48.

### Case 2

A 53-year-old male (height 163 cm, weight 60 kg) who developed dyspnea due to left pleural effusion 2 months before and underwent pleural fluid aspiration. Video-assisted empyema curettage was scheduled because empyema was not improved. The patient had dyspnea at rest due to pleural effusion and cardiac dysfunction (EF around 30%) after cardiotomy 2 years ago. Consequently, he required support with dobutamine (DOB) after admission. General anesthesia with one-lung ventilation was viable based on preoperative cardiopulmonary function, but had the possibility of induction of postoperative cardiopulmonary insufficiency. Based on a discussion with the surgeon, the surgical wounds were expected to be small and the operative time short. Therefore, sedation with DEX and regional anesthesia were chosen.

He underwent ascending aorta replacement due to acute aortic dissection (Stanford A) 23 years ago, and a Bentall procedure, ascending aortic arch replacement and coronary artery bypass graft due to aortic insufficiency and enhanced dissociative space 2 years ago.

The patient was monitored with an electrocardiogram using five electrodes, a non-invasive arterial pressure meter and pulse oximeter, and an invasive arterial pressure line placed in the right radial artery. Jackson-Rees and nasal high flows were prepared for hypoxemia during surgery. To prevent cardiac arrest, defibrillator pads were attached prior to surgery. The patient was given DOB (3 μg/kg/min) due to cardiac dysfunction before entering the operating room. Chest drainage started at 0.6 μg/kg/h DEX without a loading dose. Surgery was performed after regional anesthesia with 0.75% ropivacaine at an insertion point in the surgical field prior to surgery. Lidocaine (50 mg/h) was administered by continuous intravenous infusion to prevent a cough reflex due to thoracoscopic procedures during surgery, and fentanyl was used for pain as required. Hypotension was treated with increased DOB (3–5 μg/kg/min) and noradrenaline (NAD) (0.01 μg/kg/min), and surgery was completed without problem. The patient was controlled with oxygen of 15 L using a ventilation mask with a head band to improve adherence because of a concern about a decrease in SpO2 by one-lung ventilation during the operation. The operative time was 37 min, and the anesthetic time was 59 min. The hemorrhage volume was small and the infusion volume was 150 mL. During surgery, thoracoscopic procedures caused transient hypotension, and DOB and NAD were administered. However, DOB alone was used after surgery and postoperative hemodynamics did not differ from the preoperative state. After surgery, the patient was fully awake and returned to the ICU without oxygen supplementation. The patient was followed up without any problems. On POD 1, the patient was moved out of the ICU and had no complications due to anesthesia. He was discharged from hospital on POD 19.

## Discussion

Non-intubated VATS with epidural anesthesia for metastatic lung tumor was first performed by Vincenzo et al. in 2001, with achievement of satisfactory short- and long-term results [1]. Non-intubated VATS is used for patients with low respiratory function due to conditions such as large unilateral pleural effusion, emphysema, diffuse lung disease, multiple nodules, pericardial effusion with coexisting pleural effusion, and chronic hemothorax [2]. The surgical procedures include partial pneumonectomy and lobectomy for pneumothorax and lung tumor, and lung volume reduction surgery for emphysema [3].

The advantages of spontaneous breathing without intubation include prevention of tears caused by intubation procedures, ventilator-induced barotraumas, and atelectasis. However, intraoperative anesthetic management requires sufficient analgesia and prevention of cough reflex and body movement, which places more stress on anesthesiologists in comparison with general anesthesia. Non-intubated VATS with regional anesthesia, such as PVB and infiltration anesthesia, causes fewer complications, including unstable postoperative hemodynamics and postoperative nausea and vomiting (PONV), in comparison with general anesthesia [4]. There are no differences in complications or mortality, and pain control is good with regional anesthesia, which contributes to a decreased length of stay in the postoperative recovery unit and early ambulation and discharge from hospital [5].

PVB has a similar effect on postoperative analgesia to that of epidural anesthesia, but causes less intraoperative hypotension, and consequently has fewer complications, including urinary retention and PONV [8]. Epidural anesthesia sometimes causes severe hypotension in patients with insufficient cardiopulmonary reserve due to cardiac dysfunction and low blood volume conditions. General anesthesia was considered to be difficult for case 1 due to poor respiratory conditions; however, he underwent thoracoscopic pleural biopsy in combination with PVB while awake. PVB provides analgesia in a lateral field with local anesthetic induction in the paraspinal space. Epidural anesthesia was initially considered to be possible; however, the patient complicated with moderate AS with cardiac dysfunction and low blood volume conditions. Consequently, epidural anesthesia was excluded considering risks for hypotension by bilateral sympathetic nerve block. PVB extension can be unpredictable [9], but the patient in case 1 frequently complained of pain when the apex of the lung was touched; therefore, it was better to transfer the PVB insertion point to the head side by 1 to 2 intercostal spaces. For case 2, the operation time was expected to be short and video-assisted empyema curettage with infiltration anesthesia, rather than epidural anesthesia and PVB, which block the sympathetic nerves, was performed based on possible respiratory failure and cardiac dysfunction.

Cough reflex disturbs surgical procedures in non-intubated VATS, but can be inhibited by continuous administration of lidocaine and preoperative inhalation of 2% lidocaine, ipsilateral stellate-ganglion block and intrathoracic vagal block [6, 7]. In case 1, lidocaine was not used to avoid local anesthetic poisoning due to excessive administration of local anesthetic and PVB. However, surgery was often interrupted by cough during extensive biopsy in the thoracic cavity. In case 2, based on the experience in case 1, lidocaine was used to prevent cough with consideration of the amount of local anesthesia required for extensive curettage of the thoracic cavity. In case 2, surgery was not interrupted by cough, indicating that procedures for preventing cough reflex should have been taken in case 1.

The two patients in the cases described here had cardiac dysfunction, low blood volume, and respiratory failure before surgery, and the possibility of postoperative cardiopulmonary deterioration due to general anesthesia, although they were likely to tolerate general anesthesia. Based on these conditions, we performed non-intubated VATS under local anesthesia in these two patients. Since intraoperative immobilization was necessary for non-intubated VATS, DEX was used for light sedation. Considering preoperative cardiopulmonary insufficiency and the nature of surgery, rapid administration of DEX has risks for unexpected hypotension and oversedation-induced airway obstruction. Therefore, continuous intravenous infusion was started without preloading after the patients entered the operating room. It took approximately 1 h to start the surgery after entry into the operating room, and the sedation level was maintained from RASS-1 to − 2 without respiratory problems.

Conversion of anesthesia for non-intubated VATS to general anesthesia occurs at a rate of 0–10% for reasons including pleural adhesion, continuous hypoxemia, hypercapnia, insufficient analgesia, and hemorrhage, which suggests that conversion may be needed at any time during surgery [5, 6]. Non-intubated VATS provides surgical treatment for patients with high risks for problems with respiration who generally find it difficult to tolerate general anesthesia. Furthermore, non-intubated VATS prevents ventilator-induced barotrauma and atelectasis due to general anesthesia, which may contribute to an improved prognosis, and improves the ventilation perfusion ratio mismatch for extension of the lower lung in a lateral position, compared to intubated VATS [10]. Regarding the cardiovascular system, local anesthesia-assisted non-intubated VATS prevents general anesthesia drug-induced cardiac depression and vasodilation, leading to facile intra- and postoperative management of patients with cardiac dysfunction. For successful non-intubated VATS, it is important to ensure sufficient regional anesthesia to prevent cough reflex and body movement, which may cause risks in the surgical procedure.

### Conclusions

We safely performed non-intubated VATS in combination with PVB and infiltration anesthesia in two patients with respiratory and hemodynamic failure. Regional anesthesia techniques in non-intubated VATS are likely to be useful for patients with respiratory failure and circulatory failure, but accumulation of more case reports is necessary for establishment of evidence for the efficacy of this approach.

## Abbreviations

AS: Aortic stenosis
ASO: Arteriosclerosis obliterans
DEX: Dexmedetomidine
DOB: Dobutamine
EF: Ejection fraction
NAD: Noradrenaline
POD: Postoperative day
PVB: Paravertebral block
VATS: Video-assisted thoracic surgery
